# Oxygen consumption of gastrocnemius medialis muscle during submaximal voluntary isometric contractions with and without preceding stretch

**DOI:** 10.1038/s41598-017-04068-y

**Published:** 2017-07-05

**Authors:** F. K. Paternoster, D. Hahn, F. Stöcker, A. Schwirtz, W. Seiberl

**Affiliations:** 1 Biomechanics in Sports, Technical University of Munich, Munich, Germany; 2 Human Movement Science, Ruhr-University Bochum, Bochum, Germany; 3 School of Human Movement and Nutrition Sciences, University of Queensland, Brisbane, Australia

## Abstract

After an active muscle stretch, maintaining a certain amount of force in the following isometric phase is accompanied by less muscle activation compared to an isometric contraction without preceding active stretch at the corresponding muscle length. This reduced muscle activation might be related to reduced metabolic costs, such as the oxidative metabolism. Hence, the aim of this study was to clarify if mechanisms associated with stretch-induced activation reduction (AR) also influence oxygen consumption of voluntary activated human muscles after active stretch. Plantarflexion torque of 20 subjects was measured during 1) purely isometric and 2) active stretch contractions (26°, 60°/s), at a submaximal torque level of 30% MVC. Oxygen consumption (m$\dot{\rm{V}}$O_2_) of gastrocnemius medialis (GM) was estimated by near-infrared spectroscopy while applying arterial occlusion. Since the overall group did not show AR at GM after active stretch (p > 0.19), a subgroup was defined (n = 10) showing AR of 13.0 ± 10.3% (p = 0.00). However, for both purely isometric and active contractions m$\dot{\rm{V}}$O_2_ was the same (p = 0.32). Therefore, AR triggered by active stretch did not affect m$\dot{\rm{V}}$O_2_ of active human muscle.

## Introduction

In 1952, Abbott and Aubert made experiments on the toad sartorius muscle and found enhanced forces in the isometric phase after active stretch compared to purely isometric contractions. The final isometric muscle length as well as the muscle stimulus intensity were the same for the two conditions^1^. This finding of the so-called “residual force enhancement” (RFE) provided the basis for a continual research.

Despite the growing number of studies, the underlying mechanism(s) of RFE is (are) still unknown^2–5^. From a phenomenological perspective the muscular feature is known to be independent of stretch velocity^6^ but sensitive to the stretch amplitude^1, 7^. RFE has been proven for the entire force-length relationship^8, 9^ and has been verified in *in vivo* studies for maximum voluntary^10–12^ and submaximal voluntary contractions^13–16^, for small^10^ and large human muscles^17^ as well as for multi-joint movements^11, 14, 15^. Beside the aforementioned lack of knowledge regarding the origin of RFE, especially studies on humans performing voluntary contractions reported a discrepancy between the total number of subjects involved in a study and those who showed enhanced forces after active stretch, referred to as responder vs. non-responder phenomenon^15, 18, 19^.

In 2005, the definition of RFE was extended to submaximal contractions while keeping the applied force constant^20^. In 2005, Oskouei and Herzog found changes in the EMG signal in the isometric phase after active stretch, compared to pure isometric contractions at the same muscle length, resulting in a lower EMG signal for the isometric contraction preceded by an active stretch. Also in later work, this enhanced neuromuscular efficiency was assumed to be beneficial in terms of reduced metabolic cost during muscle contraction after active stretch^13, 21^. However, only one study directly tested this hypothesis of reduced metabolic cost after active stretch on a muscle fibre level^22^. These authors demonstrated a reduction in the ATPase activity per unit force for the isometric contraction after active stretch, compared to the purely isometric contraction for a skinned fibre from rabbit psoas muscle. They suggested that the average force per cross bridge or the engagement of a passive structure is responsible for this enhanced fibre efficiency. However, it is unclear if these results can be transferred to a complex system like *in vivo* human muscle contraction.

As one of the main methods of energy production in skeletal muscle is the oxidative metabolism, enhanced economisation triggered by active stretch contraction would positively influence the energy demands inside the muscle. A possibility to monitor processes of muscle metabolism non-invasively is provided by the use of near-infrared spectroscopy systems (NIRS). Such optical measurement systems are used in a variety of different settings, ranging from isometric to dynamic muscle contractions measuring variant muscles^23–25^. One of the first experiments calculating muscle oxygen consumption (m$\dot{{\rm{V}}}$O_2_) using light at different wavelength were done by Millikan^26^ on the cat soleus. These days, using NIRS devices in combination with arterial occlusion enables the possibility to estimate m$\dot{{\rm{V}}}$O_2_ in active *in vivo* muscles^27–29^.

Little is known regarding *in vivo* muscle oxygen consumption within the field of residual force enhancement and it is unclear if the rare information about metabolic benefits of RFE can be transferred to human muscle action. Therefore, the purpose of this study was to estimate oxygen consumption of gastrocnemius medialis (GM) during isometric, submaximal plantar flexion, with and without a preceding active stretch. The submaximal contractions were chosen because most everyday movements are based on non-maximal efforts.

## Methods

### Subjects

Twenty healthy male subjects (29 ± 4 y, 80 ± 7 kg, 183 ± 6 cm) with no history of ankle joint injuries or neurological disorders participated in the study. The subjects had a mean adipose tissue thickness of 4.0 ± 1.9 mm at the gastrocnemius medialis. The study was approved by the local Ethics Committee of the Technical University of Munich and conducted according to the declaration of Helsinki. Subjects voluntarily participated and gave written informed consent.

### Experimental Protocol

Previous to the experiment, subjects performed a training session to become familiar with the testing setup and procedure, especially to train the reproducibility of submaximal contractions at 30% maximum voluntary contraction (MVC). Biofeedback (proActive, prophysics AG, CH) was presented on a screen in front of the subjects showing the plantar flexion torque signal from the right ankle.

For further understanding of the study it is worth knowing that the starting positions represents the initial position of the active stretch contraction, whereas the reference position reflects the angular position where the parameters were assessed for the comparison of the two different contractions.

For calculation of the torque level throughout the submaximal contractions, the test session started with four maximum voluntary plantar flexion contractions, two at each position (starting position: 13.3 ± 0.4° plantar flexion, reference position: 13.0 ± 0.4° dorsiflexion. 0° defining the position when the tibia axis is perpendicular to the plantar aspect of the foot) in randomized order. To guarantee standardized conditions throughout the MVC contractions (~3 s each) the examiner gave maximum verbal encouragement. The torque curve was presented the whole time in front of the subject and the verbal starting signal was the same for all MVC contractions. In addition, the subjects were instructed to contract as fast and hard as possible.

**Figure 1:**
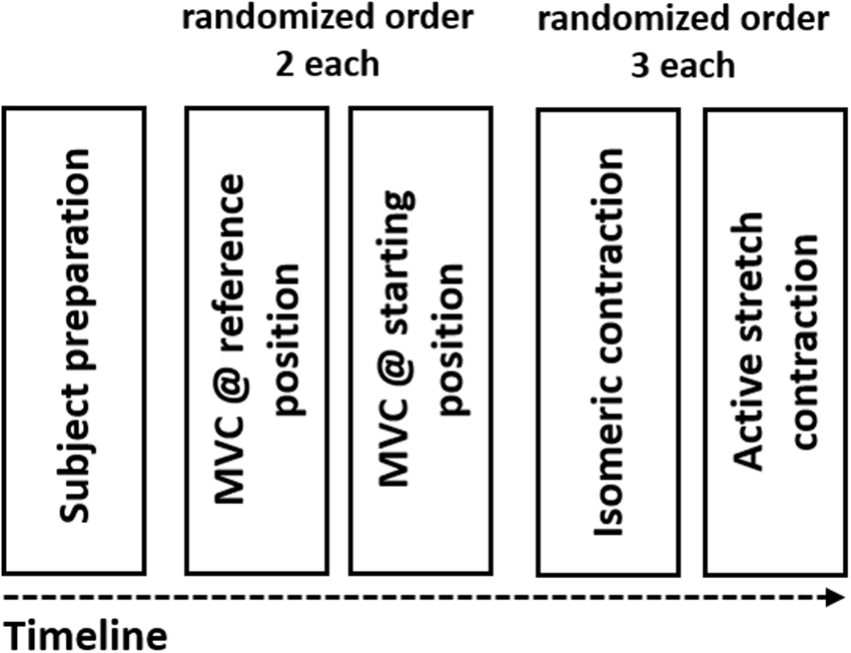
Schematic timeline of the performed experiment, measuring plantar-flexion torque. After the subject preparation, the participant did two maximum voluntary contractions (3–5 s) at the starting (13.3 ± 0.4° dorsiflexion) and reference position (13.0 ± 0.4° plantar flexion) in randomized order. These contractions were followed by either isometric (13.0 ± 0.4° plantar flexion) or active stretch contractions (Range of motion: 26.3 ± 0.4°. Angular velocity: 60°s^−1^) executed in randomized order with an activation level of 30% MVC. Rest between the different contractions was set to a minimum of 3 minutes^30^.

The maximum torque value from the two contractions at each position was defined as peak torque and subsequently was used for calculation of the submaximal target level (~30% MVC). MVC contractions were followed by three submaximal pure isometric contractions in the reference position and three active stretch (isometric-eccentric-isometric) contractions with a stretch amplitude of 26.3 ± 0.4° at an angular velocity of 60°s^−1^ (Fig. 1). The six contractions were performed in randomized order, each lasting 60 s. During active stretch contraction, a stable feedback curve of 2 s was required before the active stretch phase was initiated (Fig. 3A). Hence, as the active stretch lasted ~0.5 s, the isometric phase in the reference position was about 57.5 s for the active stretch contraction and 60 s for the pure isometric contraction. Between every contraction, subjects rested as long as individual required; but a minimum of rest was set to 3 min^30^.

### Experimental Setup

**Figure 2:**
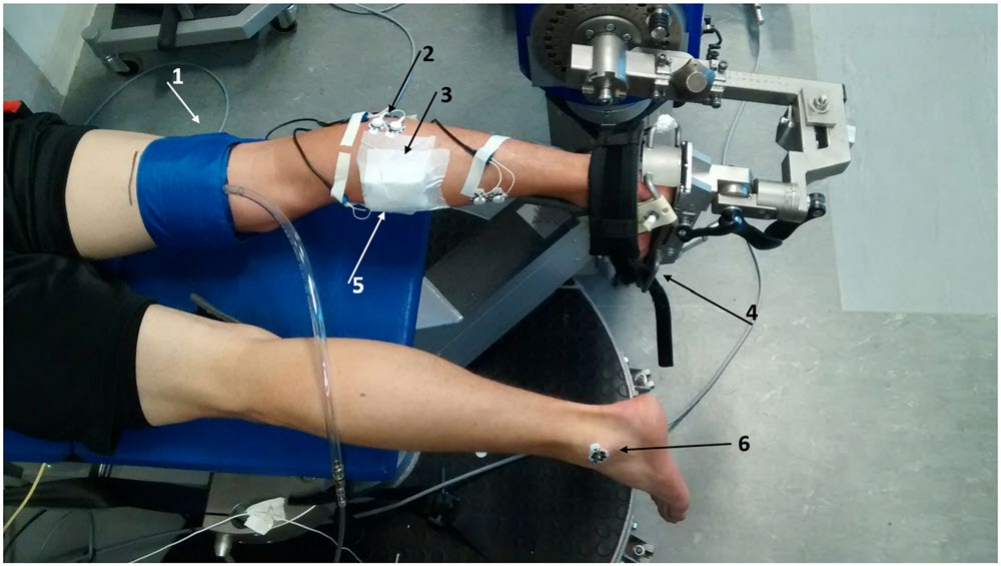
Experimental Setup. 1: Pressure Cuff. 2: EMG Electrodes. 3: Near-infrared spectroscopy device. 4: Footrest including safety belts and binding system. 5: Position of gastrocnemius medialis electrodes. 6: Reference electrode.

Plantar flexion torque was measured (1000 Hz) on a motor-driven dynamometer (IsoMed 2000, D&R Ferstl GmbH, GER). Subjects lay prone on the bench of the dynamometer. The right foot was fixed with a binding system around the instep and the heel was secured with a safety belt to avoid heel displacement during the different contractions (Fig. 2). Additionally, the subjects were fixed with shoulder pads.

Muscle activation of gastrocnemius medialis (GM), gastrocnemius lateralis (GL), soleus (SOL), and tibialis anterior (TA) were recorded (proEMG, prophysics AG, CH) with 1000 Hz (USB-6218 BNC, 16-bit, National Instruments Corporation, USA) using a wired, surface EMG system (OT Bioelecttronica, I). Skin preparation (for EMG and NIRS measurement) as well as electrode placement were done according to the SENIAM recommendations^31^. Due to the usage of a near-infrared spectroscopy system on GM, EMG electrode placement was slightly adjusted regarding the SENIAM recommendations (Fig. 2). The reference electrode was placed on the lateral malleolus of the contralateral side as the experimental setup did not allow to place it on the ipsilateral side. (Figure 2). The NIRS probe was placed on the muscle belly of GM. A constant blood volume in the lower leg was ensured via arterial occlusion. Therefore, a pressure cuff (400 mmHg, Hokanson 10D, Bellevue, WA) was placed just above the knee and was rapidly inflated (~3 s) (Fig. 2) directly prior to the onset of the submaximal contractions. To ensure the same placement throughout the different contractions the cuff position was tagged with a permanent marker. In order to avoid varying blood volumes prior to the different contraction conditions, the pressure cuff was always inflated in the reference position. After reaching the target pressure, the subjects were passively brought into the starting position for active stretch contractions, whereas for the isometric contractions a passive movement across the whole ROM was done prior to the beginning of the contraction. The pressure cuff was immediately deflated at the end of the submaximal contractions.

### Near-infrared spectroscopy

A wireless continuous-wave (CW) NIRS device (PortaMon, Artinis Medical Systems, NL) was used to estimate local oxygen consumption in GM during submaximal contractions. The interoptode distance of 40 mm as used in our setup resulted in a penetration depth of about 20 mm^32^. The wavelengths of the light source were 760 and 850 nm. Data were collected with a sample frequency of 10 Hz. The NIRS device was secured with adhesive tape and an elastic bandage to ensure the same placement throughout the test. In addition, a light-tight piece of cloth was placed around the NIRS system to avoid influence from ambient light.

CW NIRS systems can measure wavelength-specific changes in the optical density of the tissue, reflecting the tissue-oxygenation level in primarily small blood vessels^33^ using the modified Lambert-Beer law^34^. As haemo- and myoglobin are the main absorbers for light of the applied wavelength, the density changes were transformed into concentrations changes of oxyhaemoglobin (O_2_Hb) and oxymyoglobin (O_2_Mb) as well as deoxyhaemoglobin (HHb) and deoxymyoglobin (HMb). Due to the overlap in the absorption spectrum it is not possible to distinguish between haemoglobin and myoglobin proteins. Therefore, in the present study O_2_Hb and HHb represents both oxygenated and deoxygenated proteins, respectively. Since the path-length of the photons travelling through the tissue is unknown when using CW NIRS, the measurements were done using a differential path length factor of 4 for calculation of absolute concentration changes.

Beside density changes, the NIRS device provides the measurement of the tissue saturation index (TSI) which is a percentage measurement of tissue oxygen saturation and independent of near-infrared photon path length. The TSI value was used to exclude different oxygenation levels at the onset of the two contraction conditions and is calculated as

1 $$TSI={({{\rm{O}}}_{2}{\rm{H}}{\rm{b}}/({{\rm{O}}}_{2}{\rm{H}}{\rm{b}}+{\rm{H}}{\rm{H}}{\rm{b}}))}^{\ast }100.$$

Skinfold thickness was measured with a skinfold caliper (Harpenden, Baty International, GB). To obtain the adipose tissue thickness the results from the caliper measurements were divided by two^35^.

### Modelling of triceps surae muscle activity

To get a better overview across the EMG data during the force controlled setup and to account for the complexity of the m. triceps surae (TS), EMG data was weighted according to the physiological cross-sectional area and muscle volume^13^ which is thought to be directly related to maximum muscle force^36^. The weighting factors for TS (EMG_TS_) were taken from Albracht *et al*.^37^. Net EMG activation was calculated as follows:

2 $${{\rm{EMG}}}_{{\rm{TS}}}={{\rm{SOL}}}_{{\rm{measured}}}* 0.62+{{\rm{GM}}}_{{\rm{measured}}}* 0.26+{{\rm{GL}}}_{{\rm{measured}}}* 0.12$$

### Data analysis

Because the NIRS data was stored on a separate computer, signals were synchronized with an external device (PortaSync, Artinis Medical Systems, NL) and transferred to MATLAB (The MathWorks, Inc., USA) for further analysis.

**Figure 3:**
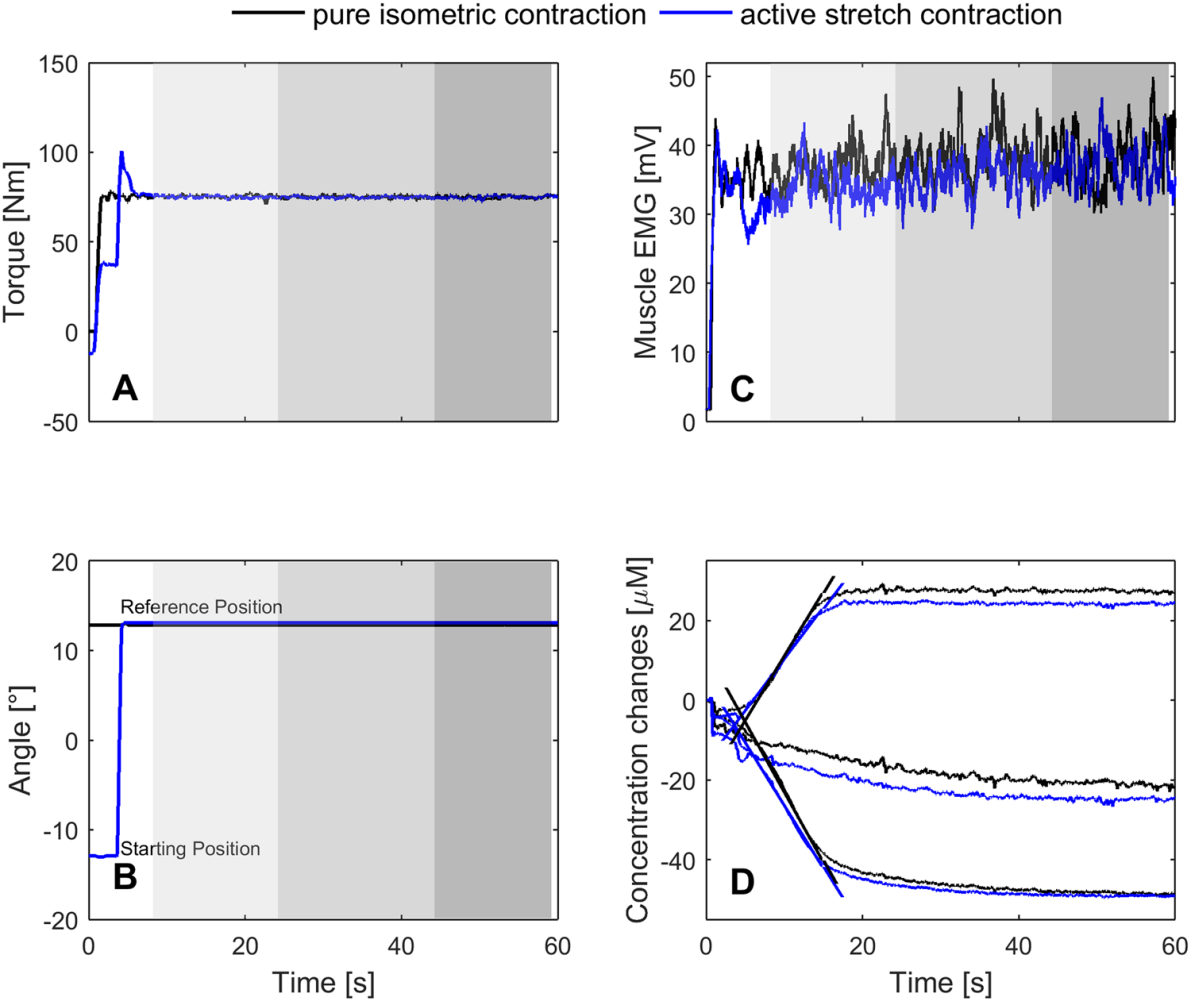
Exemplar data of measured parameters during an active stretch contraction (blue) and a pure isometric contraction (black). Different background colours in (**A**–**C**) represent three analysed time windows (Light grey: 4–20 s. Grey: 20–40 s. Dark grey: 40–55 s). A: Plantar flexion torque. B: Ankle angle. C: Muscle EMG of gastrocnemius medialis. (**D**) Near-infrared spectroscopy data. Upper-two lines represent deoxygenated haemoglobin. Mid-two lines represent total haemoglobin. Lower-two lines represent oxygenated haemoglobin. For these data the mean out of the slopes from the oxy- and deoxygenated haemoglobin of the two contraction conditions was calculated as representative of muscle oxygen consumption. Note: The time references in (**A**–**C**) are the same for both contraction conditions.

For each contraction condition, the contraction with the lowest standard deviation from the biofeedback torque target curve was chosen for final processing. Angle and torque were filtered using a 5 Hz Low pass zero-lag filter. EMG signals were band-pass filtered (10–500 Hz) with a second order Butterworth zero-lag filter, rectified and smoothed (0.5 s moving average). For statistical analysis regarding differences of the two contraction conditions, each 60 s lasting contraction was divided into 3 sections starting after the end of stretch (T1: 4–20 s, T2: 20–40 s, T3: 40–55 s) (Fig. 3A–C). Since the start of each contraction was defined when the torque value exceeded 10 Nm, the time-points of the analysis section from the active stretch contraction could be assigned to the pure isometric contraction. Thereby we can ensure that the duration of the muscle activation was the same at each analysis section for each condition. For torque and angle data the mean of each section was calculated whereas for EMG data the integral for each analysis section was computed using the trapezoidal numerical integration function of MATLAB.

Activation reduction for each analysis section was calculated as

3 $$AR=(1-{{\rm{E}}{\rm{M}}{\rm{G}}}_{\text{active}{\rm{\_}}\text{stretch}}/{{\rm{E}}{\rm{M}}{\rm{G}}}_{\text{pure}{\rm{\_}}\text{isometric}})\times 100.$$

Hence, positive values indicate activation reduction.

The baseline values of the NIRS data, calculated as mean over a 30 s time period prior to start of the contractions, were subtracted from the signal in a first step. Afterwards the data were smoothed using a Loess filter (span 10%) as the differentiation of the raw NIRS signal was too noisy for calculation of peak m$\dot{{\rm{V}}}$O_2_.

The peak m$\dot{{\rm{V}}}$O_2_ value was needed for an iteration method defining the linear slope of the O_2_Hb and HHb signal with a goodness of fit for r^2^ > 0.99. The mean of the slopes was taken as representative of m$\dot{{\rm{V}}}$O_2_^27^. The calculation of the offset corrected slopes started at the beginning of the contraction, defined when torque exceeded 10 Nm. The slopes of O_2_Hb and HHb were normalized to the individual maximum value at the end of each contraction, to make our results comparable between subjects. Analogous to the calculation of the activation reduction, the relation between m$\dot{{\rm{V}}}$O_2_ from the pure isometric and the active stretch contraction was calculated as

4 $${{\rm{m}}\dot{{\rm{V}}}{\rm{O}}}_{2} \% =(1-{\text{m}\dot{{\rm{V}}}{\rm{O}}}_{2\_\text{active}\_\text{stretch}}/{\text{m}\dot{{\rm{V}}}{\rm{O}}}_{2\_\text{pure}\_\text{isometric}})\times 100.$$

Thus, positive values represent reduced oxygen consumption.

In addition, for the pure isometric as well as for the active stretch contraction, the tissue saturation index was calculated at the onset of the contraction (TSI_start_iso_, TSI_start_as_) as well as just after end of stretch (TSI_stretch_iso_, TSI_stretch_as_) for both contraction conditions.

### Statistics

Data were tested concerning normal distribution using the Shaprio Wilk test. If normality was confirmed, a 2 × 3 (condition × time) repeated measure ANOVA was calculated and the results were corrected if sphericity was violated using Greenhouse-Geisser correction. In the case of only comparing two means, we used a student’s t-test for paired groups. Once normality was rejected Friedmans’s ANOVA was calculated. In the case of a significant result, a Wilcoxon test was used for further analysis. The alpha level was set to p < 0.05 (two-sided) and analysis was performed using IBM SPSS 23 software (SPSS for Windows, US).

### Data availability

The dataset generated and analysed during the current study are available from the corresponding author on reasonable request.

## Results

Detailed descriptive data can be found in Tables 1 and 2. Data are presented as mean ± standard deviation.

**Table 1:** Mean and standard deviation (±) of all subjects during pure isometric (iso) and active stretch (as) contractions at three analysed windows.

Overall Group (n = 20)	T1_iso	±	T1_as	±	T2_iso	±	T2_as	±	T3_iso	±	T3_as	±
**Torque [Nm]**	75.5	11	75.6	11	75.5	10.9	75.5	10.9	75.5	10.9	75.6	11.0
**Angle [**°**]**	13.0	0.5	13.0	0.3	13.0	0.5	13.0	0.4	13.0	0.4	13.0	0.3
**EMG**_**TS**_ [mV*s]	2.0	1.6	1.9	1.5	2.6	2.1	2.5	2.0	2.0	1.6	2.0	1.6
GM [mV*s]	3.1	2.3	3.0	2.4	3.9	3.1	3.8	2.4	2.8	2.2	2.8	2.2
GL [mV*s]	1.8	2.3	1.7	2.1	2.4	1.8	2.4	2.6	2.0	1.5	1.9	1.8
SOL [mV*s]	1.5	1.2	1.5	1.1	2.1	1.8	2.1	1.6	1.7	1.4	1.7	1.4
TA [mV*s]	0.4	0.3	0.4	0.3	0.5	0.3	0.5	0.3	0.5	0.4	0.4	0.3
*TSI_**_start**_ **[%]**	69.7	4.2	70.7	3.8								
**TSI_**_stretch**_ **[%]**	67.8	3.7	68.8	3.5								

T1 = 4–20 s after stretch. T2 = 20–40 s after stretch. T3 = 40–55 s after stretch. Torque = Plantar flexion torque. Angle = Angular position at the reference position. EMG_TS_ = Triceps surae. GM = Gastrocnemius medialis. GL = Gastrocnemius lateralis. SOL = Soleus. TA = Tibialis anterior. Torque and angle data represent mean data. GM, GL, SOL and TA represent integrated data. *TSI = Tissue saturation index just prior to the onset of the contraction. **TSI__stretch_ = Tissue saturation index just after the end of stretch.

**Table 2:** Mean and standard deviation (±) of all subjects during pure isometric (iso) and active stretch (as) contractions at T1.

Subgroup (n = 10)	T1_iso	±	T1_as	±
Torque [Nm]	75.8	8.7	75.5	9.0
Angle [°]	12.9	0.1	13.0	0.2
EMG_TS_ [mV*s]	**1.9**	0.9	**1.8**	0.9
GM [mV*s]	**3.3**	1.6	**2.9**	1.6
GL [mV*s]	1.7	0.9	1.7	1.3
SOL [mV*s]	1.4	0.8	1.4	0.7
TA [mV*s]	0.4	0.2	0.5	0.2
*TSI__start_ [%]	71.8	3.5	72.4	3.9
**TSI__stretch_ [%]	68.8	3.81	70.3	3.5

T1 = 4–20 s after stretch. Torque = Plantar flexion torque. Angle = Angular position at the reference position. GM = Gastrocnemius medialis. GL = Gastrocnemius lateralis. SOL = Soleus. TA = Tibialis anterior. Torque and angle data represent mean data. GM, GL, SOL and TA represent integrated data *TSI__start_ = Tissue saturation index just prior to the onset of the contraction. **TSI__stretch_ = Tissue saturation index just after the end of stretch. Bold numbers represent significant differences between contraction conditions at specific analysed time window.

### Overall group

Maximum NIRS values (O_2_HB_iso = 34.2 ± 10.7 µM; O_2_Hb_ecc = 35.5 ± 9.9 µM; HHb_iso = 24.1 ± 8.4 µM; HHb_ecc = 24.2 ± 9.1 µM) used for normalization of m$\dot{{\rm{V}}}$O_2_ were statistically not different between contraction conditions (O_2_Hb: t (19) = −1.27, p = 0.22. HHb: t (19) = −0.36, p = 0.72). The estimated muscle oxygen consumption (m$\dot{{\rm{V}}}$O_2_) showed no differences between the two conditions (t (19) = −1.71, p = 0.10). The tissue saturation index (TSI) at the onset of the contraction showed a trend towards higher values for the active stretch condition (t (19) = −2.00, p = 0.06), the same was true for the parameter TSI__stretch_ (t (19) = −2.05, p = 0.05) (Table 1).

Friedmans’s ANOVA indicated significant differences in overall angular positions of the ankle (χ^2^ (5) = 15.66, p = 0.01). Wilcoxon post-poc tests did not identify differences of angle between contraction conditions at specific analyses windows (T1: T = 55, p = 0.62, T2: T = 53, p = 0.52, T3: T = 99, p = 0.82). Repeated measures ANOVA showed no differences regarding the torque values for factor condition (F (1, 19) = 0.24, p = 0.88), time (F (1.35, 25.70) = 0.40, p = 0.60), and interaction of condition and time (F (2, 38) = 0.70, p = 0.50).

Regarding the EMG activity the modelled triceps surae activity (EMG_TS_) (χ2 (5) = 72.77, p = 0.00) as well as GM (χ^2^ (5) = 70.26, p = 0.00), GL (χ^2^ (5) = 45.71, p = 0.00), SOL (χ^2^ (5) = 75.80, p = 0.00) and TA (χ^2^ (5) = 68.37, p = 0.00) showed significant results. Calculating Wilcoxon tests, neither EMG_TS_ (T1: T = 58, p = 0.62, T2: T = 72, p = 0.52, T3: T = 92, p = 0.82) nor the activity of GM (T1: T = 80, p = 0.35, T2: T = 70, p = 0.19, T3: T = 92, p = 0.63), GL (T1: T = 72, p = 0.22, T2: T = 71, p = 0.20, T3: T = 78, p = 0.31), SOL (T1: T = 91, p = 0.60, T2: T = 102, p = 0.91, T3: T = 75, p = 0.26) and TA (T1: T = 97, p = 0.77, T2: T = 82, p = 0.39, T3: T = 75, p = 0.26) showed differences between contraction conditions at the three different analysed time-windows.

A positive correlation was found between m$\dot{{\rm{V}}}$O_2_% and AR (r = 0.69, p = 0.001), whereas there was no correlation between TSI_stretch_ and m$\dot{{\rm{V}}}$O_2_% (r = −0.18, p = 0.46) (Fig. 5).

### Subgroup

To test the hypothesis whether an activation reduction in the post-isometric phase after an active stretch affects muscle oxygen consumption, a subgroup showing activation reduction (AR) in the GM during the first analysed time window (T1) was defined. 50% (n = 10) of the overall group fulfilled this criteria. T1 (4–20 s after stretch) was chosen as the plateau region of the O2Hb and HHb signal was reached about 15–20 s after contraction start (Fig. 3D). Hence, this gives us the opportunity to ensure that activation reduction was present in those subjects and to connect activation reduction with estimated oxygen consumption.

Statistical analysis at T1 showed no differences between contraction conditions regarding torque (t (9) = −1.27, p = 0.35) and EMG of GL (t (9) = −0.40, p = 0.70), SOL (t (9) = −1.27, p = 0.31) and TA (t (9) = −1.27, p = 0.36), TSI_start_ (t (9) = −1.27, p = 0.52) and TSI_stretch_ (t (9) = −2.00, p = 0.076). For the ankle angle there was a significant but irrelevant difference at T1 (T = 6, p = 0.03) between the pure isometric contraction and the active stretch contraction (12.9 ± 0.1° vs. 13.0 ± 0.2° dorsiflexion).

**Figure 4:**
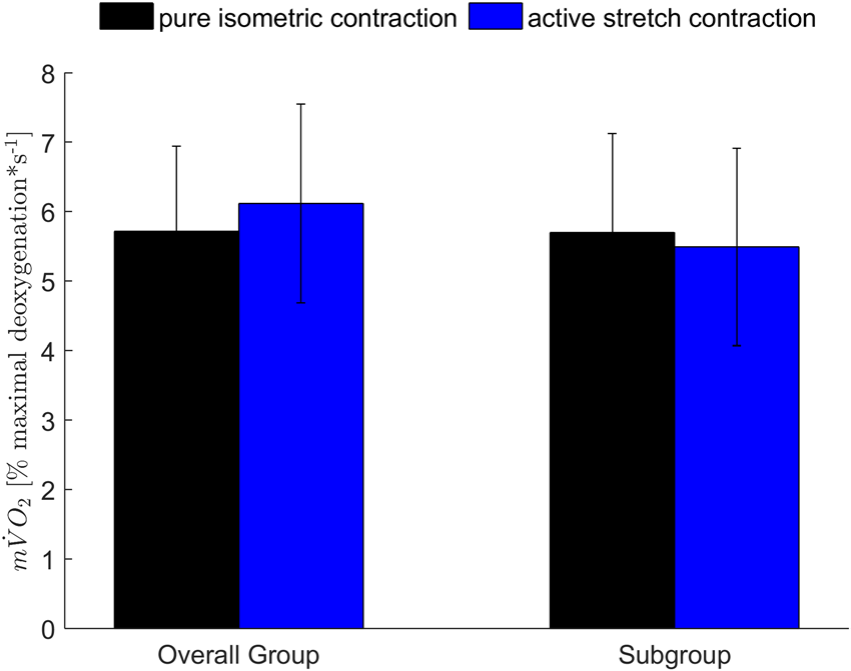
Results (mean ± standard deviation) for the estimated oxygen consumption for the overall subject group (n = 20) and the subgroup (n = 10). No significant differences were found between isometric and active stretch contraction.

There was a significant difference for the muscle activity of EMG_TS_ (t (9) = 4.76, p = 0.00) and GM (t (9) = 4.12, p = 0.00). Showing an AR after active stretch of 7.7 ± 4.8% and 13.0 ± 10.3% compared with the pure isometric contraction, respectively. However, m$\dot{{\rm{V}}}$O_2_ showed no significant difference between contraction conditions (t (9) = 1.05, p = 0.32, Fig. 4).

## Discussion

The purpose of the present study was to clarify if AR during an isometric contraction following an active stretch is associated with a reduced muscle oxygen consumption in comparison to a pure isometric contraction without preceding active stretch. This purpose is based on the repeatedly reported finding of enhanced neuromuscular efficiency after active stretch compared to a pure isometric contraction^13, 20, 38^. Although never measured for an *in vivo* muscle, it was concluded from these results that the observed reduced AR in the isometric phase after active stretch is accompanied by reduced metabolic cost^13, 21^.

As a requirement for testing our hypothesis, two criteria must be fulfilled for the analysis of m$\dot{{\rm{V}}}$O_2_ regarding activation reduction in GM in our setup:

1. Subjects must be responders regarding activation reduction after active stretch at GM.
2. Activation reduction must occur in the GM concerning the detection of reduced m$\dot{{\rm{V}}}$O_2_. At the same time, the remaining investigated muscles of the triceps surae should not show an enhanced muscle activation during the active stretch compared to the pure isometric contraction. This is necessary to exclude a compensation of AR at GM by muscle redundancy^39^.

The overall subject group did not fulfil the first criterion. There was no AR in the target muscle GM even so the second criterion was fulfilled as GL and SOL showed no difference when comparing the isometric phase after active stretch with the pure isometric condition. Likewise, m$\dot{{\rm{V}}}$O_2_ showed no difference between contraction conditions (Fig. 4). To overcome the problem of muscle redundancy^39^ during a specific task, Seiberl *et al*.^13^ modelled the overall EMG-activity of the involved muscles by calculating a net muscle activity according to the physiological cross-sectional area and the muscle volume of the involved muscles. However, applying this approach to the triceps surae did not show enhanced neuromuscular efficiency in terms of activation reduction after active stretch. Hence, our data is different regarding the existence of activation reduction compared to previously published data of muscles involved in human locomotion^13, 38^. Especially the number of non-responders is higher than reported for comparable work: 20–30% in Seiberl *et al*.^13^, ~30% in Oskuei and Herzog^18^, ~10% in Tilp *et al*.^19^. In our study, 50% of the subjects showed no activation reduction after active stretch for GM. Despite numerus reports that especially during submaximal contractions only a part of the subjects is showing reduced activation or enhanced torque after active stretch in comparison to a pure isometric contraction^15, 18, 19^, by now no existing explanation gives a valid and satisfying answer for these observations. Fibre type distribution, subject specific individual threshold regarding the level of muscle activation or the lack of certain muscle physiological abilities are among the discussed theories^40^. Unfortunately, our data does not allow to draw any conclusions regarding that phenomenon.

For testing whether the estimated oxygen consumption is different between a pure isometric and an isometric contraction preceded by an active stretch, a subgroup was chosen according to the predefined criteria.

50% of our participants fulfilled the first criteria showing AR in the GM at T1. T1 (4–20 s after stretch) was chosen as the time point when the NIRS signals reached the plateau phase was between 15 to 20 s after contraction start (Fig. 3D). Thus, this time window allows direct connection of activation reduction with estimated oxygen consumption. Standardized condition for this subgroup were given regarding torque production. Ankle angle showed a significant difference at T1. As the calculated angle difference between the contraction conditions was less than 0.1°, the discrepancy is supposed to have no influence on the interpretation. For the other parts of the triceps surae we found no differences between contraction conditions, therefore fulfilling the second criteria. The same was true for the antagonistic muscle TA. Hence, the subgroup satisfied both criteria as well as the standardization criteria between the contraction conditions and showed an activation reduction of 13% in the gastrocnemius medialis and about 7% for the modelled net EMG-activity data at T1. For the active stretch contraction, Oskouei and Herzog^18, 20^ showed an activation reduction for the thumb ranging between 7 and 11%, Altenburg *et al*.^38^ found a reduction of about 10% and the data of Seiberl *et al*.^13^ revealed a AR of about 8% both obtained for the knee extensor muscle.

To guarantee the same oxygen status of the investigated tissue, the tissue saturation index was taken just prior to the onset of the contraction. The results showed no difference and a tissue saturation of about 72% for both contractions which is in the range of previously published work^41, 42^. For the subgroup, oxygen consumption estimated for GM showed a reduction of 3.2% for the isometric phase after active stretch compared to the pure isometric contraction but was statistically not different. This is in contrast to the study published from Joumaa and Herzog^22^ who found a reduced ATPase activity per unit force in the isometric phase after an active stretch compared to a pure isometric contraction for a skinned rabbit psoas muscle fibre. They assumed, inter alia, that an enhanced force per cross-bridge is associated with the results. This is line with Altenburg *et al*.^38^ suggesting a derecruitment of active motor units after active stretch. Referring to de Ruiter *et al*.^27^ proposing a reduced m$\dot{{\rm{V}}}$O_2_ as indicator towards the number of force generating cross-bridges, our results do not support the theories from Joumaa and Herzog^22^ and Altenburg *et al*.^38^.

**Figure 5:**
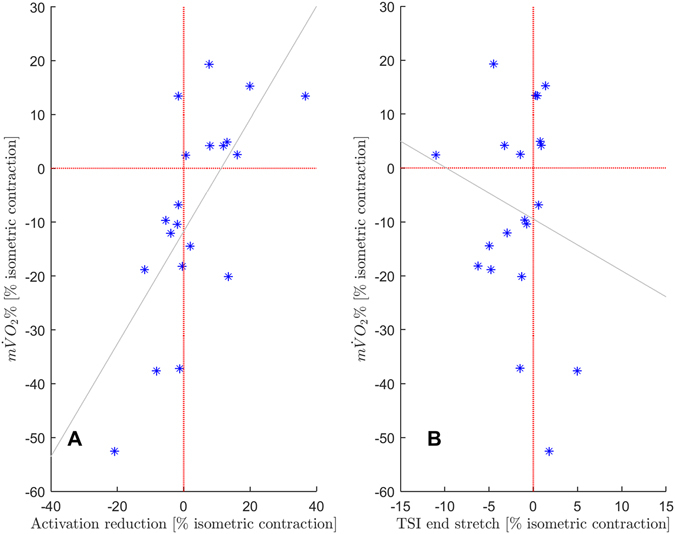
(**A**) Scatter plot between activation reduction (x-axis) and oxygen consumption (m$\dot{{\rm{V}}}$O_2_) (y-axis). Positive values on the y- and x-axis are attributed to activation reduction (AR) and reduced m$\dot{{\rm{V}}}$O_2_ compared to pure isometric contraction. Data shows a significant correlation between m$\dot{{\rm{V}}}$O_2_% and activation reduction. (r = 0.69, p = 0.001). (**B**) Scatter plot between TSI value at the end of stretch (x-axis) and oxygen consumption (m$\dot{{\rm{V}}}$O_2_%) (y-axis). Positive values on the y- and negative values on the x-axis are attributed to reduced m$\dot{{\rm{V}}}$O_2_% and enhanced TSI values after end of stretch compared to pure isometric contraction. TSI at the end of stretch was not correlated to m$\dot{{\rm{V}}}$O_2_ in following isometric states. (r = −0.18, p = 0.46). Note: Each symbol represents one subject.

The rate of oxygen consumption (5.6% maximum deoxygenation*s^−1^) is in accordance with previous published data from Kooistra *et al*.^43^ and Ruiter *et al*.^27^, investigating quadriceps femoris at 30% of maximum torque capacity. Although there was a strong positive correlation between muscle activation and m$\dot{{\rm{V}}}$O_2_ (Fig. 5A), statistics revealed no difference regarding the oxygen consumption between an active stretch contraction and a pure isometric contraction. Hence, for our subgroup the results are contrary to published literature where reduced muscle activation was associated with lower m$\dot{{\rm{V}}}$O_2_, when comparing different levels of muscle activation^43–45^.

Praagman *et al*.^46^ showed a high positive correlation between muscle activation and m$\dot{{\rm{V}}}$O_2_ ranging between r = 0.81 and r = 0.94 for m. biceps brevi and m. brachiroadialis. These are higher values than in our study (Fig. 5A, r = 0.69, p = 0.001). Nevertheless, there seems to be a relation between activation reduction caused by an active stretch and oxygen consumption of the investigated muscle. Reasons of possible mechanism triggered during an active stretch are still under debate. The idea of a stretch-loaded (active) spring within the sarcomere would help to explain reduced metabolic demands of a muscle to maintain a certain amount of force. However, this is highly speculative and way beyond methodologically based conclusions of this study.

Alterations of blood-volume while applying arterial occlusion can sometimes occur due to an ongoing redistribution of blood within the limb and has been described in literature as an increase in tHb^23, 44, 47^. Such an increase in tHb is associated with an ongoing re-oxygenation of the area under the investigated muscle. A constant blood volume under the optode will result in mirrored graphs for O_2_Hb and HHb, while tHb, calculated out of the sum of O_2_Hb and HHb nearly stays constant. A re-oxygenation might mask changes in the NIRS signal regarding the resulting slopes representing muscle oxygen consumption. Visually inspecting the graphs of tHb we found no re-oxygenation of blood volume during the individual trials (Fig. 3D) which suggests a fully occluded lower leg in our study. In addition, the pressure of the cuff used to establish arterial occlusion is in the range of previous studies^27, 28^ regarding the lower limb. Another point to discuss is the possible influence of the active stretch regarding the NIRS region of interest. Despite inflating the pressure cuff always in the reference position and performing the same amount of ankle joint motion for each condition (passive shortening and an active stretch for dynamic task; passive shortening-stretch prior to the pure isometric contraction), we cannot exclude that an active stretch had different effects on the underlying tissues in comparison to a pure isometric contraction. To clarify a possible influence, we additionally calculated the TSI values for both contraction types at the time point “end of the stretch” to evaluate if the region of interest had the same oxygen status. Results revealed a slightly enhanced (p = 0.05) TSI directly after stretch for the active stretch condition (68.8 ± 3.5%) compared to the pure isometric contraction (67.8 ± 3.5%) at the corresponding time point. Correlation between m$\dot{{\rm{V}}}$O_2_ and TSI at the end of stretch provided no additional information (r = −0.18, p = 0.46, Fig. 5B). Therefore, the initial contraction phase until the time point end of stretch does not affect the two contraction conditions in a different manner. Consequently, estimated differences regarding m$\dot{{\rm{V}}}$O_2_ during the isometric phase after active stretch compared with the pure isometric contraction can be attributed to mechanisms triggered by active stretch.

In general, using near-infrared devices has always some limitations. The estimation of m$\dot{{\rm{V}}}$O_2_ via near-infrared spectroscopy is limited because it primarily reflects concentration changes in small blood vessels, such as the capillary or arteriolar and venular beds^33^. Therefore, no direct conclusion can be drawn about deep parts of muscle tissue. In this context, adipose tissue thickness is known to additionally affect the measurement of m$\dot{{\rm{V}}}$O_2_, as the path of the light is different in adipose tissue compared with muscle tissue. Hence, a high amount of subcutaneous fat will result in an underestimation of m$\dot{{\rm{V}}}$O_2_^35^. However, as this study is based on a repeated within-subject measure design, effects of tissue thickness variability should not have any influence on the presented results.

To our knowledge, this was the first study in the field of residual force enhancement testing the assumption of a stretch-induced energetic optimization in *in vivo* human muscles using near-infrared spectroscopy. Our data did not confirm reduced metabolic cost in terms of oxygen consumption, as there was no difference between active stretch condition and pure isometric contraction. As muscle redundancy could have influenced our data, for future studies it is suggested to focus on less complex muscles in first instance to assess the basic phenomenological relations.
